# Functional MRI Mapping of Human Meniscus Functionality and its Relation to Degeneration

**DOI:** 10.1038/s41598-020-59573-4

**Published:** 2020-02-12

**Authors:** Sven Nebelung, Lisa Dötsch, Dhaval Shah, Daniel Benjamin Abrar, Kevin Linka, Matthias Knobe, Philipp Sewerin, Johannes Thüring, Christiane Kuhl, Daniel Truhn

**Affiliations:** 1 Department of Diagnostic and Interventional Radiology, University Hospital Aachen, Aachen, Germany; 2 Institute of Diagnostic and Interventional Radiology, University Hospital Düsseldorf, Düsseldorf, Germany; 3 Department of Continuum and Materials Mechanics, Hamburg University of Technology, Hamburg, Germany; 4 Clinic for Orthopaedic and Trauma Surgery, Cantonal Hospital Luzern, Luzern, Switzerland; 5 Department and Hiller-Research-Unit for Rheumatology, University Hospital Düsseldorf, Düsseldorf, Germany; 6 Institute of Computer Vision and Imaging, RWTH University Aachen, Aachen, Germany

**Keywords:** Translational research, Diagnostic markers, Magnetic resonance imaging, Cartilage, Biomedical engineering

## Abstract

Meniscus pathology may promote early osteoarthritis. This study assessed human meniscus functionality (i.e. its response to loading) *ex vivo* based on quantitative T1, T1ρ, and T2 mapping as a function of histological degeneration and loading. Forty-five meniscus samples of variable degeneration were harvested from the lateral meniscus body region of 45 patients during total knee arthroplasties. Samples underwent serial mapping on a 3.0-T MRI scanner (Achieva, Philips) using a force-controlled and torque-inducing compressive loading device. Samples were measured at three loading positions, i.e. unloaded, loaded to 2 bar (compression force 37 N) and 4 bar (69 N). Histology (Pauli classification) and biomechanics (Elastic Modulus) served as references. Based on histology, samples were trichotomized as grossly intact (n = 14), mildly degenerative (n = 16), and moderate-to-severely degenerative (n = 15) and analyzed using appropriate parametric and non-parametric tests. For T1, we found loading-induced decreases in all samples, irrespective of degeneration. For T1ρ, zonal increases in intact (apex) and decreases in degenerative samples (base) were found, while for T2, changes were ambiguous. In conclusion, force-controlled loading and serial MR imaging reveal response-to-loading patterns in meniscus. Zonal T1ρ response-to-loading patterns are most promising in differentiating degeneration, while T1 and T2 aren’t clearly related to degeneration.and may provide an imaging-based indication of functional tissue properties.

## Introduction

Acute and chronic meniscus pathologies are frequent clinical entities^1^. Due to its decisive role in load bearing, load transmission, load dissipation and in providing joint stability, congruity and lubrication^2^, meniscus integrity in form and function is of utmost importance to the knee joint’s long-term health^3^. Meniscus functionality, which is the tissue’s ability to function properly, i.e. to disperse loads and reduce friction, is heavily dependent on its extracellular matrix properties. Collagen fibers, primarily type-I, define the tissue’s tensile strength and shock-absorbing properties, while proteoglycans contribute to compressive strength by upholding swelling pressure^2,4,5^. Lately, therapeutic efforts have been aimed at preserving and restoring the damaged meniscus secondary to trauma or degeneration^6,7^. This is of particular relevance as meniscus and cartilage pathologies are closely interrelated. Consequently, meniscus damage and loss are key features of and risk factors for developing osteoarthritis (OA)^8^. The discussion on whether meniscus pathologies are a cause or consequence of knee OA is ongoing^9,10^.

Yet common consensus prevails that morphological meniscus defects, i.e. surface breakdown and tissue tearing, are the consequence of degenerative changes of the extracellular matrix^11–13^, and contribute to the evolution of OA by altering load distribution and transmission to the adjacent articular cartilage. Hence, detecting such degenerative changes early is necessary in therapeutic efforts to preserve the meniscus and prevent or delay the onset of early OA^14,15^.

Due to its non-invasiveness, superior soft tissue contrast and absence of ionizing radiation, Magnetic Resonance Imaging (MRI) is clearly the most powerful and versatile imaging method of contemporary clinical medicine. Clinical-standard morphological MRI is the modality of choice in the evaluation of pathologies in and around the knee joint with high diagnostic accuracy in the assessment of gross meniscus pathologies^16^. However, evaluation is subjective and solely based on morphological aspects such as surface integrity and intra-tissue signal intensities. MRI is also limited when detecting smaller lesions and changes prior to surface breakdown^17,18^. Quantitative MRI (qMRI, synonymous with functional MRI) techniques such as T2, T1 and T1ρ mapping provide spatially resolved measures of tissue (ultra)structure and composition beyond mere morphology and have been applied to assess meniscus in health and disease^4,13,19,20^. T1ρ and T2 characteristics in meniscus have been studied extensively, whereas data on T1 characteristics are sparse. While increases in T1ρ and T2 in the presence of OA and meniscus lesions have been reported^20,21^, these parameters’ substantial intra- and inter-individual variability only allows differentiation of histological extremes, i.e. intact versus severely degenerated tissue^13^. In imaging studies, meniscus functionality refers to the loading-induced intra-tissue adaptations and their imaging correlates and has been evaluated by simultaneous qMRI mapping and axial loading^22,23^. Studying the posterior horn of the medial meniscus, Calixto *et al*. found significant loading-induced decreases in T1ρ and T2 in OA knees and no such changes in control knees^22^. For the meniscus body, the same group observed significantly larger changes in T1ρ and T2 in the medial meniscus in OA versus non-OA knees^23^. Quantifying the tissue’s response to loading by measuring the changes in T1ρ (Δ_T1ρ_), T2 (Δ_T2_) and -possibly- T1 (Δ_T1_) as surrogate parameters of functionality therefore provides innovative imaging biomarkers of load transmission and, possibly, its failure in (early) OA.

As standard values of Δ_T1ρ_, Δ_T2_, and Δ_T1_ in human meniscus are lacking, the present study aimed i) to assess the response to standardized loading of human lateral meniscus samples (body region) on the basis of qMRI mapping techniques, ii) to correlate the loading-induced changes to histological and biomechanical reference measures, and iii) to subsequently define the response-to-loading patterns of human meniscus in health and disease. The underlying research question was: What is the physiological and pathological response to loading of human meniscus (as assessed by Δ_T1ρ_, Δ_T2_, and Δ_T1_ and as controlled by histology)? Our hypotheses were that i) zonal changes in Δ_T1ρ_, Δ_T2_, and Δ_T1_ are demonstrated in response to standardized loading of meniscus as an indication of adaptive intra-tissue changes, and ii) the responses to loading thus discernible are distinctly different in grossly intact vs. early degenerative vs. moderate-to-severely degenerative meniscus.

## Results

### General findings

All 45 samples underwent complete MRI measurements and subsequent histological and biomechanical reference characterization. In all samples, sufficient tissue thickness was available for these analyses.

**Figure 1:**
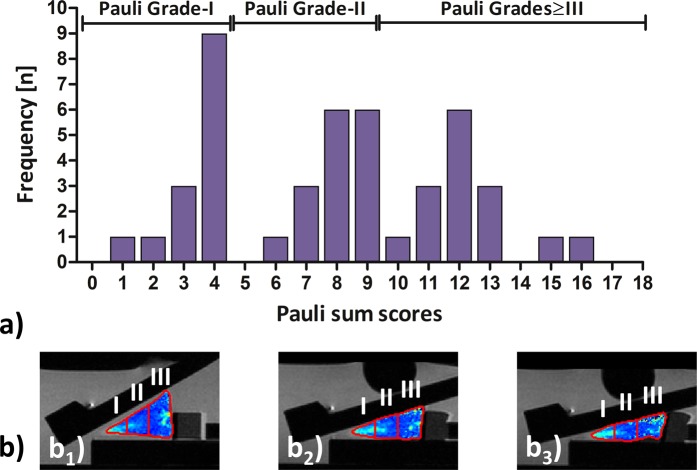
Distribution of Pauli sum scores (**a**) and sample-specific segmentation routines as a function of increasing loading (**b**). After sample allocation, 14, 16, and 15 samples constituted the Pauli Grade-I, Pauli Grade-II, and Pauli Grades ≥ III subgroups (**a**). The lateral meniscus body sample as shown in Fig. 3 is displayed across the different loading positions (b_1_-b_3_). After manual segmentation of sample outlines, the samples’cross-sectional areas were automatically partitioned into thirds along the mediolateral dimension, i.e. the apex (I), intermediate (II), and base regions (III). Color-coded T2 maps were overlaid onto the corresponding PD-weighted morphological images. Scale bar as in Fig. 3d.

Histologically, samples displayed a wide range of different manifestations with Pauli sum scores ranging from 1 to 16 (Fig. 1a). Accordingly, n = 14 samples constituted the grossly intact subgroup (Pauli Grade-I), n = 16 samples the mildly degenerative subgroup (Pauli Grade-II), and n = 15 samples the moderate-to-severely degenerative subgroup (Pauli Grades ≥ III). Mean Pauli sum scores were 3.4 ± 0.9 (Pauli Grade-I), 8.0 ± 0.9 (Pauli Grade-II), and 12.3 ± 1.5 (Pauli Grades ≥ III).

Elastic Modulus at strains 20% and 80% was not significantly different between the subgroups: strain 20%: 15.5 ± 10.1 MPa (Pauli Grade-I), 17.8 ± 12.5 MPa (Pauli Grade-II), 16.2 ± 9.9 MPa (Pauli Grades ≥ III) (p = 0.893); strain 80%: 351.4 ± 74.3 MPa (Pauli Grade-I), 343.4 ± 70.4 MPa (Pauli Grade-II), 353.5 ± 67.1 MPa (Pauli Grades ≥ III) (p = 0.919).

Unloaded, we did not find significant degeneration-dependent differences in the various regions-of-interest (ROIs) for T1 or T1ρ. For T2, however, we determined significant differences in the apical zone with significantly lower T2 medians in Pauli grade-I than in Pauli grades-II (and higher) samples (p = 0.004). Overall, qMRI parameter values tended to be highest in Pauli Grades ≥ III samples and in the apical zone. Unloaded absolute qMRI parameters are given in Table 1.

**Table 1:** Absolute quantitative MRI parameter values of lateral meniscus body samples in response to sequential loading.

		T1					T1ρ					T2				
		δ0	δ1	δ2	p-value (§)	post-hoc	δ0	δ1	δ2	p-value (§)	post-hoc	δ0	δ1	δ2	p-value (§)	post-hoc
all (n = 45)	Entire Sample	696 (641–785)	605 (560–642)	554 (525–586)	**<0.001**	*, *, *	46.7 (40.1–56.6)	46.6 (43.7–54.0)	47.2 (43.4–54.5)	0.841		24.7 (22.8–27.7)	24.2 (22.5–26.0)	23.0 (21.9–24.5)	**0.001**	ns, *, ns
	Apex (I)	882 (747–976)	820 (695–939)	761 (630–867)	**<0.001**	ns, *, *	53.3 (40.8–69.1)	58.6 (54.1–67.8)	54.1 (47.7–66.6)	0.041		29.9 (22.8–37.2)	28.7 (23.0–35.6)	24.6 (21.9–29.4)	**<0.001**	ns, *, *
	Intermediate (II)	626 (572–745)	529 (487–627)	471 (436–537)	**<0.001**	*, *, *	41.7 (34.3–51.6)	44.6 (39.9–49.6)	43.4 (40.8–49.0)	0.052		21.0 (19.6–24.5)	20.6 (19.4–23.1)	20.2 (18.8–22.4)	0.023	
	Base (III)	661 (615–731)	509 (474–597)	487 (452–543)	**<0.001**	*, *, *	48.3 (42.3–57.1)	46.2 (40.6–49.2)	44.7 (39.9–52.2)	0.022		24.8 (22.8–27.6)	23.6 (22.5–26.0)	23.5 (22.0–25.8)	0.001	ns, *, ns
Pauli Grade-I (n = 14)	Entire Sample	678 (590–779)	574 (504–634)	558 (518–577)	**<0.001**	*, *, ns	43.3 (36.9–53.2)	46.2 (44.0–51.4)	47.9 (43.4–52.4)	0.135		24.0 (22.1–26.2)	23.3 (21.8–25.2)	23.0 (21.3–24.1)	0.145	
	Apex (I)	780 (600–911)	713 (599–901)	702 (620–818)	0.526		43.6 (35.5–49.1)	58.6 (55.2–63.2)	55.0 (48.3–63.3)	**0.001**	*, *, ns	23.3 (19.8–28.1)	23.0 (21.6–28.3)	22.8 (21.6–25.4)	0.395	
	Intermediate (II)	593 (511–686)	522 (448–607)	465 (425–505)	**<0.001**	*, *, ns	34.8 (31.6–39.2)	44.9 (41.0–48.2)	44.7 (41.5–46.5)	**<0.001**	*, *, ns	20.2 (19.0–21.1)	20.3 (19.4–22.5)	20.2 (18.7–21.9)	0.424	
	Base (III)	661 (641–750)	513 (483–562)	499 (448–546)	**<0.001**	*, *, ns	48.2 (41.2–56.3)	46.1 (40.6–48.0)	43.9 (40.0–48.1)	0.395		26.2 (23.1–28.9)	23.6 (22.4–26.7)	22.6 (22.0–26.1)	0.135	
Pauli Grade-II (n = 16)	Entire Sample	691 (647–781)	588 (555–655)	579 (520–607)	**<0.001**	*, *, ns	47.0 (40.2–59.5)	48.0 (44.3–54.7)	53.2 (43.6–59.9)	0.611		25.2 (23.0–27.9)	24.6 (22.7–27.9)	23.5 (22.5–26.3)	0.020	
	Apex (I)	887 (748–1077)	877 (703–962)	837 (607–988)	0.015		63.3 (43.8–85.2)	59.7 (56.5–74.7)	58.3 (48.8–67.9)	0.305		30.1 (23.8–40.1)	29.8 (24.3–38.0)	26.0 (21.4–31.1)	**0.002**	ns, *, *
	Intermediate (II)	624 (569–718)	532 (468–588)	482 (436–535)	**<0.001**	*, *, ns	42.2 (35.3–51.0)	44.2 (39.7–49.6)	42.9 (40.7–54.0)	0.210		25.3 (22.9–26.8)	23.8 (22.5–27.4)	25.0 (23.5–26.8)	0.368	
	Base (III)	676 (624–754)	528 (432–606)	511 (457–578)	**<0.001**	*, *, ns	45.1 (40.0–58.9)	47.9 (41.8–52.0)	48.0 (41.8–59.7)	0.646		26.8 (24.5–27.9)	26.4 (24.1–28.4)	27.0 (25.9–29.0)	0.646	
Pauli Grades ≥ III (n = 15)	Entire Sample	751 (653–806)	613 (593–653)	543 (531–579)	**<0.001**	*, *, *	52.3 (41.6–58.4)	46.4 (41.7–51.9)	45.7 (41.9–53.2)	0.155		25.3 (23.2–29.0)	24.3 (23.2–25.6)	23.0 (21.5–24.4)	0.085	
	Apex (I)	914 (824–1013)	860 (727–929)	782 (633–853)	**<0.001**	ns, *, *	65.5 (50.2–69.6)	56.9 (44.5–67.4)	51.3 (45.0–69.2)	0.819		31.0 (29.0–38.7)	32.1 (26.7–38.0)	28.6 (24.6–32.4)	**<0.001**	ns, *, *
	Intermediate (II)	694 (615–803)	569 (515–649)	489 (449–567)	**<0.001**	ns, *, *	49.7 (37.9–55.4)	45.5 (39.6–52.6)	43.4 (39.5–49.5)	0.449		23.7 (19.5–27.5)	21.4 (19.6–25.3)	19.7 (18.2–23.1)	0.017	
	Base (III)	628 (603–718)	505 (481–572)	468 (449–508)	**<0.001**	*, *, ns	49.7 (42.5–58.9)	41.9 (39.7–48.2)	41.0 (38.5–49.0)	**<0.001**	*, *, ns	23.5 (21.7–27.3)	23.6 (22.5–24.8)	22.0 (20.1–23.8)	0.031	
p-values (†)	Entire Sample	0.410	0.204	0.605			0.184	0.470	0.242			0.257	0.325	0.461		
	Apex (I)	0.090	0.249	0.375			0.026	0.326	0.616			0.004 (*, *, ns)	0.043	0.086		
	Intermediate (II)	0.041	0.257	0.538			0.024	0.911	0.981			0.532	0.762	0.844		
	Base (III)	0.699	0.988	0.393			0.817	0.300	0.107			0.324	0.855	0.005 (ns, ns, *)		

Data are given as median (interquartile range) [ms]. Regions-of-interest (ROIs) include the entire sample as well as the apical (I), intermediate (II) and base (III) zones. Histological Pauli grade-related differences for the distinct ROIs at the successive loading positions were assessed using the Kruskal-Wallis test (^†^), while loading-related longitudinal differences were assessed using the Friedman test followed by Dunn’s post-hoc test (§). For the post-hoc test results, statistically significant differences are given in the order δ_0_ vs δ_1_, δ_0_ vs δ_2_, and δ_1_ vs δ_2_ (§) or Pauli-grade I vs. Pauli-grade II, Pauli-grade I vs. Pauli-grade III, and Pauli-grade II vs. Pauli-grade III (†) and are printed in **bold type**. Significant differences are indicated by “*” and non-significant differences by “ns”. δ_0_ refers to the unloaded configuration, while δ_1_ and δ_2_ refer to loaded configurations at 2 bar (compression force 37.1 N) and 4 bar (69.1 N).

### Qualitative changes in response to loading

Qualitatively, loading-induced changes were variable across the samples. Meniscus samples were subject to substantial compression and deformation under loading. Consequently, the physiological wedge shape underwent considerable flattening with increased loading.

**Figure 2:**
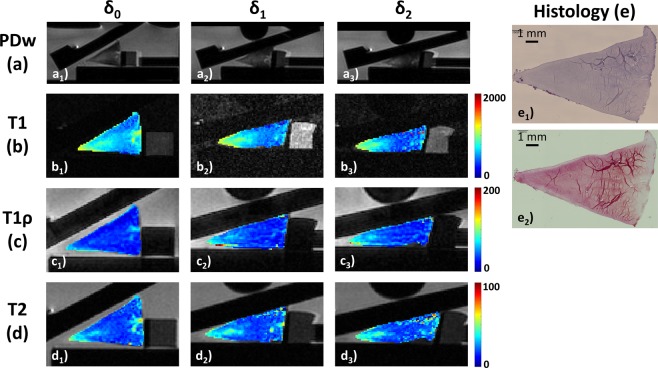
Serial morphological images (Proton Density-weighted [PDw], (**a**) and T1 (**b**), T1ρ (**c**), and T2 (**d**) maps of histologically intact human meniscus as controlled by histology (**e**) and displayed as a function of loading. Serial PDw (**a**), T1 (**b**), T1ρ (**c**), and T2 maps (**d**) of meniscus samples from the lateral body region are displayed at increasing loading intensities (δ_0_: unloaded [a_1_–d_1_]; δ_1_: loaded to compression force of 37 N [a_2_–d_2_]; δ_2_: loaded to 69 N [a_3_–d_3_]). Following manual segmentation, color-coded qMRI parameter maps were overlaid onto the corresponding morphological images. Color codes on the right extend from 0–2000 (T1), 0–200 (T1ρ), and 0–100 ms (T2). The corresponding histological sections are shown (**e**) after Hematoxylin-Eosin (e_1_) and Safranin-O staining (e_2_). Histologically, this sample demonstrated slight fraying of the meniscus apex zone but otherwise smooth surfaces without fraying or fibrillation (score 1), normal cell distribution (score 0), diffuse foci of degenerated extracellular matrix (score 1), and moderate staining intensity for proteoglycans (score 2). Pauli sum score 4 (Pauli grade-I). Of note, focal calcifications of the tibial meniscus surface are only secondary histological findings and not relevant to the histological scoring.

**Figure 3:**
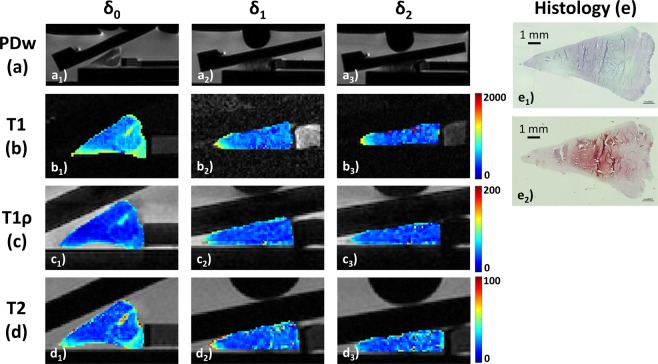
Serial morphological images (**a**) and qMRI parameter maps (**b**–**d**) of histologically mildly degenerative human meniscus as controlled by histology (**e**) and displayed as a function of loading. As in Fig. 2, serial PDw (**a**), T1 (**b**), T1ρ (**c**), and T2 maps (**d**) are displayed at increasing loading intensities. Histologically (**e**), this sample had mild-to-moderate superficial fibrillations and undulations (score 5), normal cell distribution (score 0), largely confluent foci of degenerated extracellular matrix alongside unorganized collagen fibres and fraying (score 2), and moderate staining intensity for proteoglycans (score 2). Pauli sum score 9 (Pauli grade-II). Figure details as in Fig. 2.

**Figure 4:**
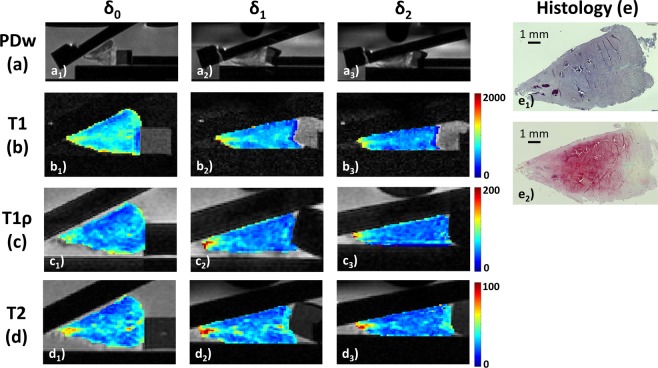
Serial morphological images (**a**) and qMRI parameter maps of histologically moderately degenerative human meniscus as controlled by histology (**e**) and displayed as a function of loading. As in Figs. 2 and 3, serial PDw (**a**), T1 (**b**), T1ρ (**c**), and T2 maps (**d**) are displayed at increasing loading intensities. Histologically (**e**), this sample demonstrated signs of severe surface disintegration and disruption, particularly at the tibial meniscus surface with apical tearing (score 7), diffusely hypercellular regions (score 1), bands of hyaline degeneration and fraying of collagen fibres (score 2), and still moderate staining intensity for proteoglycans (score 2). Pauli sum score 12 (Pauli grade-III). Focal calcifications at the apical and intermediate zones. Figure details as in Figs. 2 and 3.

In Pauli Grade-I samples, only the apical and intermediate zones underwent slight changes, i.e. decreases in T1, and increases in T1ρ and T2, while the base zone was largely unaltered (Fig. 2). In Pauli Grade-II and ≥ III samples, the apical zone underwent largest changes. In some samples, signal characteristics became more homogeneous so that pre-existent signal hyperintensities gradually disappeared (Fig. 3), while in other samples, focal signal hyperintensities that were not well appreciable in the unloaded configuration became more prominent under loading, in particular at the apical zone (Fig. 4). All samples, irrespective of degeneration, were characterized by substantial loading-induced decreases in T1 throughout the entire cross-sectional area, whereas changes in T1ρ or T2 characteristics were less uniform. In Figs. 2–4, loading-induced changes in the morphological (Figs. 2a and 4a) and qMRI parameter maps (T1 [Figs. 2b–4b], T1ρ [Figs. 2c–4c], and T2 [Figs. 2d–4d]) and their histological references (Figs. 2e–4e) are visualized.

### Quantitative changes in response to loading

Quantitatively, loading-induced adaptations as detailed above were reflected by several changes: First, mean pixel numbers decreased significantly from 801 ± 237 (unloaded, δ_0_) to 718 ± 263 (loading position 1, δ_1_), and 604 ± 236 (loading position 2, δ_2_) (p < 0.001, entire sample). Second, we found distinct changes in T1, T1ρ and T2 in response to loading. Table 1 summarizes absolute qMRI parameter values as a function of increasing loading and histological Pauli Grades, while Table 2 gives the respective relative changes.

**Table 2:** Relative changes in quantitative MRI parameter values of lateral meniscus body samples in response to sequential loading, i.e. Δ_1_ and Δ_2_ [%].

		T1			T1ρ			T2		
		Δ1	Δ2	p-value (§)	Δ1	Δ2	p-value (§)	Δ1	Δ2	p-value (§)
all (n = 45)	Entire Sample	−15.1 ± 7.0	−20.5 ± 8.5	**0.001**	0.9 ± 13.1	3.6 ± 17.6	0.425	0.4 ± 20.2	−6.0 ± 12.8	0.077
	Apex (I)	−6.5 ± 19.0	−9.6 ± 12.9	0.371	14.8 ± 30.7	9.9 ± 30.1	0.443	0.9 ± 13.5	−9.1 ± 16.3	**0.002**
	Intermediate (II)	−15.5 ± 10.2	−24.4 ± 11.3	**<0.001**	9.0 ± 22.2	11.4 ± 29.2	0.671	1.4 ± 30.2	−4.9 ± 17.9	0.234
	Base (III)	−22.1 ± 9.2	−26.7 ± 10.5	0.033	−7.3 ± 14.9	−26.7 ± 10.5	0.508	−0.9 ± 24.0	−4.0 ± 18.0	0.492
Pauli Grade-I (n = 14)	Entire Sample	−16.4 ± 6.4	−20.4 ± 9.6	0.211	7.0 ± 13.5	8.6 ± 21.2	0.818	−2.4 ± 8.4	−3.6 ± 15.3	0.792
	Apex (I)	−0.3 ± 14.2	−3.0 ± 13.9	0.612	38.4 ± 27.7	29.4 ± 31.5	0.431	7.2 ± 15.0	2.5 ± 13.1	0.381
	Intermediate (II)	−12.6 ± 8.2	−21.1 ± 10.7	0.027	23.2 ± 25.2	27.3 ± 38.2	0.743	0.3 ± 10.1	−1.3 ± 18.0	0.782
	Base (III)	−24.2 ± 9.1	−27.1 ± 11.3	0.460	−6.5 ± 15.5	−7.3 ± 19.3	0.899	−5.1 ± 12.1	−5.7 ± 20.3	0.926
Pauli Grade-II (n = 16)	Entire Sample	−14.6 ± 7.5	−18.6 ± 8.1	0.163	2.6 ± 12.8	6.7 ± 15.9	0.427	5.8 ± 30.8	−4.7 ± 8.7	0.200
	Apex (I)	−5.2 ± 13.1	−8.8 ± 12.5	0.441	12.2 ± 31.4	4.3 ± 30.0	0.472	2.0 ± 12.5	−13.8 ± 18.0	0.007
	Intermediate (II)	−17.0 ± 10.4	−23.9 ± 11.6	0.090	5.8 ± 17.9	7.5 ± 19.4	0.798	7.7 ± 48.8	−4.0 ± 11.6	0.356
	Base (III)	−21.9 ± 10.6	−25.1 ± 11.7	0.427	−2.8 ± 14.7	4.2 ± 20.7	0.281	4.8 ± 35.8	1.7 ± 16.5	0.752
Pauli Grades ≥ III (n = 15)	Entire Sample	−14.4 ± 7.1	−22.8 ± 8.0	**0.005**	−6.5 ± 9.7	−4.5 ± 13.5	0.641	−2.9 ± 12.0	−9.6 ± 13.9	0.164
	Apex (I)	−13.6 ± 25.8	−16.5 ± 9.0	0.683	−4.4 ± 15.3	−2.4 ± 19.7	0.760	−6.2 ± 10.1	−14.8 ± 11.8	0.040
	Intermediate (II)	−16.6 ± 11.8	−28.2 ± 11.2	0.012	−0.8 ± 17.5	0.6 ± 22.9	0.853	−4.3 ± 11.7	−9.2 ± 23.0	0.475
	Base (III)	−20.2 ± 7.9	−27.9 ± 8.9	0.020	−12.7 ± 13.6	-12.2 ± 13.0	0.916	−2.9 ± 15.6	−8.3 ± 16.9	0.374
p-value (†)	Entire Sample	0.702	0.387		0.014	0.090		0.412	0.400	
	Apex (I)	0.160	0.015		**<0.001**	0.009		0.022	**0.004**	
	Intermediate (II)	0.456	0.241		0.008	0.035		0.544	0.493	
	Base (III)	0.525	0.743		0.177	0.044		0.496	0.285	

Data are given as mean ± standard deviation. Δ_1_ (or Δ_2_) were calculated by relating the absolute qMRI parameter values at loading position δ_1_ (or δ_2_, respectively) to the unloaded configuration δ_0_. Histological Pauli grade-related differences for the distinct regions-of-interest at the successive loading positions were assessed using one-way ANOVA tests (†), while differences between Δ_1_ and Δ_2_ were assessed using unpaired Student’s t-tests (§). Statistically significant are printed in **bold type**. Please refer to Table 1 for details on loading positions and sample allocation.

For T1, we found significant decreases in all samples, irrespective of the histological Pauli Grade (e.g. all samples, entire ROI: δ_0_: 696 (641–785) ms, δ_1_: 605 (560–642) ms, δ_2_: 554 (525–586) ms, p < 0.001). Decreases were larger at the meniscus base and related to loading intensity, i.e. larger loading induced larger decreases (e.g. all samples, entire ROI: Δ_1_: −15.1 ± 7.0%, Δ_2_: −20.5 ± 8.5%, p = 0.001). Significantly larger relative changes were found for the intermediate zone and the entire sample.

For T1ρ, we made numerous distinctly different observations. Even though mean relative changes were not significantly different for the entire samples (all samples, entire ROI: Δ_1_: 0.9 ± 13.1%, Δ_2_: 3.6 ± 17.6%, p = 0.425), they were distinctly different in the meniscus zones. First, we found -by trend- loading-induced decreases at the base zone. These were significant in Pauli Grades ≥ III samples (base ROI: δ_0_: 49.7 (42.5–58.9) ms, δ_1_: 41.9 (39.7–48.2) ms, δ_2_: 41.0 (38.5–49.0) ms, p < 0.001). Second, we observed discrepant and significantly different changes at the apical zone. Here, T1ρ values increased in Pauli Grade-I and -II samples (Pauli Grade-I samples, apex ROI: δ_0_: 43.6 (35.5–49.1) ms, δ_1_: 58.6 (55.2–63.2) ms, δ_2_: 55.0 (48.3–63.3) ms, p = 0.001), while they decreased non-significantly in Pauli Grades ≥ III samples (Pauli Grades ≥ III samples, apex ROI: δ_0_: 65.5 (50.2–69.6) ms, δ_1_: 56.9 (44.5–67.4) ms, δ_2_: 51.3 (45.0–69.2) ms, p = 0.819). Changes in the intermediate zone were similar. Third, the amplitude of changes was not clearly associated with loading intensity.

For T2, we found ambiguous zonal loading responses, even though overall, decreases were more prevalent than increases. Even though in all samples, significant decreases were found with increasing loading (all samples, entire ROI: δ_0_: 24.7 (22.8–27.7) ms, δ_1_: 24.2 (22.5–26.0) ms, δ_2_: 23.0 (21.9–24.5) ms, p = 0.001), further zonal analysis revealed that these significant changes were exclusive to the apical zone with differences being significant in all samples (p < 0.001), Pauli Grade-II samples (p = 0.002) and Pauli Grades ≥ III samples (p < 0.001).For the other zones, however, changes were not significant. By trend, relative changes tended to be larger at higher loading intensity, even though significant differences were only found for the apical zone of all samples (all samples, apex ROI: Δ_1_: 0.9 ± 13.5%, Δ_2_: −9.1 ± 16.3%, p = 0.002).

We did not find significant correlations between histological measures and qMRI parameters, while the correlations between biomechanical parameters and qMRI parameters, i.e. between the Elastic Modulus at strains 20% and at 80% and T1, T1ρ, and T2 at δ_0_, δ_1_ and δ_2_ were inverse and largely significant. More specifically, inverse significant correlations were found for all unloaded qMRI parameters and Elastic Modulus at both strains (−0.67 ≤ Spearman’s ρ ≤ −0.43; p ≤ 0.003). While these findings were confirmed for δ_1_ (except for T1ρ [δ_1_]: ρ = −0.38, p = 0.01), correlations were not significant anymore for δ_2_ (except for T1 [δ_2_]: ρ = −0.47, p = 0.001). Please see Supplementary Table 1 for more details.

## Discussion

The most important findings of this study are 1) lateral human meniscus body samples undergo distinctly different zonal changes in T1, T1ρ and T2 in response to loading and 2) these changes are associated with histological degeneration but not with biomechanical properties, i.e. Elastic Modulus. More specifically, in histologically grossly intact meniscus, the physiological response to loading is defined by decreases in T1, divergent changes in T1ρ with increases at the apex and decreases at the base, and ambiguous changes in T2. In severely degenerative meniscus however, the pathological response to loading is characterized by homogenous decreases in T1ρ throughout the tissue, while for T1 and T2, similar changes as for the intact samples were found. Across all samples and histological subgroups, substantial inter-individual variability could be determined. Even though we employed statistical measures and tests that decrease sensitivity to outliers, the substantial variability certainly prevented clearer statistical inferences and biological conclusions. Except for T2 in the apical zone, we could not find any significant degeneration-related differences for any ROI in the unloaded configuration. In all samples, we found that the apical zone (by trend) had the highest and the intermediate zone the lowest T1, T1ρ, and T2 values. This finding is well in line with recent *in-vivo* and *in-vitro* studies that reported regional and zonal variability in human meniscus due to tissue heterogeneity in structure, composition and biomechanical properties^4,13,22–24^.

In our study, loading induced morphological deformation and flattening of the wedge shape and, correspondingly, significant decreases in the compressed samples’ pixel numbers, which is indicative of sufficient sample pressurization. Within the confines of our study’s *in-vitro* design, the loading intensity may be considered grossly representative of the *in-vivo* situation. The applied compressive forces of 38 N (δ_1_) and 69 N (δ_2_) were actuated on meniscus samples of standard length (15 mm) and variable width (mean: 8.4 mm)^25^, resulting in a mean sample surface area of 126 mm² and mean pressure levels of 0.30 MPa (δ_1_) and 0.55 MPa (δ_2_), respectively. For the lateral meniscus body region, such pressures are well in line with those experienced during level walking (ca. 0.5 MPa)^26^, even though peak pressures may be considerably higher during unlevel walking, running or stair climbing. Thus, our loading regime is representative of static loading configurations as in prolonged one- or two-leg standing. Within the premise of this study’s *in-vitro* setting, we consider the inclusion of two different loading regimens (alongside the unloaded configuration) to provide a thorough understanding of the loading-induced intra-tissue adaptations and to indicate if (and to what extent) variations in loading intensities are related to variations in tissue functionality as assessed by qMRI parameters.

The observed changes in the T1, T1ρ, and T2 maps are grossly reflective of the morphological changes. Overall, the samples’ responses to loading were variable. For T1, we found consistent loading-induced decreases throughout all ROIs of all histological Pauli Grades. For cartilage, T1 relaxation has been linked to tissue hydration^27^, while for meniscus, the exact structural and/or compositional correlate is yet unknown. The loading-induced decreases are most likely due to water redistribution within the tissue, its partial loss and relative increases in the solid matrix constituents. Decreases in T1 were larger with more intense loading, in particular in the intermediate zone, which may be explained by the fact that this zone is biomechanically most compliant^28^. Mean overall decreases in T1 tended to be larger at the meniscus base, which is plausible, too, given the higher water content^4^ and the abundant amount of loose vascularized connective tissue^29^. Under loading, T1 values decreased in all samples, irrespective of degeneration, which indicates the dominant contribution of water to T1 relaxation that most likely overpowers the contribution of the biochemical variations in collagen and/or proteoglycans^4,30^. Also, MRI measurements were performed after extended sample storage in medium which may have increased tissue hydration beyond the constitutively high water content.

Loading-induced changes in T1ρ were less homogeneous. While we found decreases at the meniscus base in all samples, changes at the meniscus apex and -to a lesser extent- the intermediate zone were significantly different in the distinct Pauli Grade subgroups. In Pauli Grade-I samples, T1ρ values were increased, while in Pauli Grades ≥ III samples, they were decreased. Similar to T1, the consistent decreases in T1ρ at the meniscus base are most likely secondary to the fact that T1ρ relaxation is dominated by extracellular water, especially in advanced degeneration^4^. Consequently, degeneration-related changes in the *a-priori* heterogeneous nature of the tissue are responsible for the discrepant changes at the apical (and intermediate) zones. Under loading, the tight collagen network is compressed and condensed. Intuitively, one would speculate that T1ρ values are decreased because of more restricted spin motion. In fact, T1ρ values were increased in Pauli Grade-I samples, which is in line with previous *in-vivo* data^23^. As T1ρ probes the low-frequency interactions of the tissue’s macromolecules and bulk water^31^, physiological adaptations of the structurally intact matrix, i.e. changes in collagen fibre anisotropy, restrictions in water redistribution and upheld swelling pressure, may have led to these increases. Nonetheless, the significantly different loading responses may indicate sufficient load transmission (in Pauli Grade-I samples) and its failure (in Pauli Grades ≥ III samples), thereby corroborating earlier findings^22,23^, even though the exact biophysical correlates of these changes remain unclear.

For T2, the response-to-loading patterns were ambiguous and not related to histological degeneration. In meniscus, T2 is widely considered to be sensitive to water interactions and related to content, orientation and anisotropy of the collagen fibers^19^. Loading-induced decreases were more prevalent than increases, which is primarily indicative of changes in water distribution and content^4^. Consequently, stronger loading was associated with larger changes in T2, at least for the apical zone. Here, loading induced significant decreases in T2 in all samples except for Pauli grade-I samples, which is an indication of more pronounced degeneration-dependent changes in the apical zone. Due to its lack of intrinsic ability for self-repair, the inner zone is considered particularly prone to permanent tissue damage^32^, and consequently, degenerative tears usually extend from the inner zone to the periphery^33^. This clinical observation was confirmed in our study as tissue fraying and tearing in more degenerative samples usually originated from the apical zone.

Surprisingly, we did not find any significant differences in the biomechanical properties of the histological subgroups. As earlier reports on the relationship of meniscal degeneration and biomechanical properties are conflicting^34,35^, this finding is only partially in line with the pertinent literature. Potential reasons for this discrepancy relate to differences in study design, reference evaluation, sample numbers, degeneration severity, harvesting locations and biomechanical testing protocols. In their comparative evaluation of medial and lateral menisci, Katsuragawa *et al*. included six meniscus pairs each from OA and non-OA knee joints^34^. OA menisci were harvested from TKAs because of OA involving (at least) the medial compartment, while non-OA menisci came from knee joints without a previous history of OA as confirmed macroscopically. For biomechanical evaluation, standardized cylindrical specimens were prepared from the anterior horn and subjected to confined compression testing to determine aggregate modulus and permeability (as measures of the solid and fluid components’ contributions, respectively). Although histological work-up was performed, degeneration severity was only assessed qualitatively. The authors did not find any significant differences in the biomechanical parameters for lateral menisci from OA versus non-OA knees, while for medial menisci, they determined large differences indicating considerable softening in degenerative medial menisci. In contrast, Fischenich *et al*. found progressive decreases in biomechanical properties with increasing tissue degeneration^35^. The authors included 24 medial and lateral meniscus pairs from TKAs, sectioned them into anterior and posterior regions and performed i) indentation relaxation tests to determine the instantaneous and equilibrium compressive moduli and ii) tension testing to determine tensile modulus. Meniscal degeneration was assessed macroscopically based on inspection of gross tissue morphology, while histology was not performed. While tensile modulus was largely retained throughout degeneration, significant degeneration-dependent decreases in instantaneous and equilibrium compressive moduli were found. Whatever the exact relation of meniscal degeneration and biomechanical properties, fluid flow seems to predominate over matrix composition in determining the compressive properties of meniscus^7^, which suggests that even (histologically) moderate-to-severely degenerative meniscus upholds viscoelastic capacities to withstand compressive loading, at least when quantified by qMRI mapping techniques. This is in line with our findings that suggest that the structural and/or compositional correlates of T1 and T2 (and their loading-induced changes) are not significantly different across the different grades of histological degeneration. In contrast, zonally discrepant changes in T1ρ, i.e. significant increases at the apex in grossly intact samples and significant decreases at the base in moderate-to-severely degenerative samples, indicate that the correlates of T1ρ undergo degenerative processes that may be detected by functional imaging techniques. Moreover, the underlying tissue model/theory used to determine the mechanical properties of the meniscus samples and their assumptions are highly relevant. In the present study, we used a traditional two-parameter exponential model^36^ that is common in modelling approaches of nonlinear properties of fibril-reinforced tissues in tension such as meniscus^35,37–39^. Nonetheless, alternative models such as the linear biphasic theory^40^ have also been applied before to quantify mechanical properties of meniscus^34,41^.

This study has several limitations. First, our study’s *ex-vivo* setting limits overall transferability to the *in-vivo* setting. Alongside differences in tissue hydration due to sample storage, additional factors such as temperature, culture conditions, preparation procedure, and sample position and orientation as well as the fact that we performed the MRI measurements in standard saline solution may alter relaxation characteristics and consecutive response-to-loading patterns^42^. Nonetheless, in a clinically relevant basic research context, this study comprehensively assessed degeneration-related response-to-loading patterns in human meniscus. For once, measurements were performed on a clinical MRI scanner (and clinically applicable sequence parameters) and on samples from a clinical population representative of the entire continuum of health and disease. Meanwhile, the increasing interest in the association of joint imaging and joint mechanics (with its traditional focus on articular cartilage) has produced a number of MRI-compatible loading devices that are intended to realize the clinical translation of stress MRI techniques. Loading of patients’ knee joints (and menisci) is based on suspending weights via dedicated pulley systems^43–47^ or direct compressive loading along the leg axis^48–50^. In practical terms, further *in-situ* and *in-vivo* studies assessing meniscus functionality on the basis of such loading devices and quantitative MRI techniques are necessary to corroborate our study’s findings of the partial relatedness of loading-induced qMRI parameter changes and (histological) degeneration.

Second, the compressive loading device does only partially emulate physiological loading. While the experimental setup provides indications of the intra-tissue changes secondary to compressive loading that goes through the meniscus, its reflection of the complex biomechanical environment during loadbearing *in vivo* (that involves tensile and shear stresses^2,26,51^) is limited. Secondarily, the meniscus undergoes complex motion and adaptations, thereby deforming and displacing the perimeniscal soft tissues^51,52^. Another related aspect involves dynamic contact mechanics across the joint that change during activities of daily living, e.g. gait and stair climbing, and has considerable effects on resultant peak contact stresses of meniscus and cartilage^26^. Despite these limitations, our study’s implementation of standardized loading of meniscus samples *in vitro* is a basic requirement to realize reproducible and comparable loading regimes across sample cohorts at optimized image resolution and signal-to-noise ratio. Nonetheless, for more complex biomechanical evaluations, *in-situ* and *in-vivo* configurations are critical. Moreover, we deliberately chose an elastic polyvinylsiloxane inlay to circumferentially confine the meniscus samples. Under loading, the inlay was deformed as a sign of effective load transmission. This, however, increases the complexity of the subsequent biomechanical analyses, e.g. in finite element simulations. Third, even though sample size allowed for sound statistical analyses and valid inferences, it was limited, not least due to the study’s single-institution design. At this stage, it remains speculative -in particular when considering the qMRI parameters’ substantial statistical variability- if larger sample sizes might have brought about less equivocal findings. Fourth, as meniscus properties and their imaging correlates demonstrate considerable zonal and regional variability^4,13,22,23^, samples were harvested from the lateral meniscus body region only for the sake of topoanatomic consistency. Accordingly, our findings are only reflective of this region and may be different for the anterior and posterior regions of the lateral meniscus, let alone the medial meniscus. Fifth, samples were only harvested from total knee arthroplasties, which raises the question of our sample cohort’s representativity when it comes to truly healthy samples. In this regard, future studies should include alternative sample sources with a focus on young non-OA donors to be more representative of the entire spectrum of health and disease. Another related aspect concerns the semiquantitative histological scoring used as reference standard. Histological signs of degeneration across one sample may be variable, thereby rendering mean (subcomponent) scores potentially unrepresentative of focal alterations in matrix structure and composition that, however, may substantially affect qMRI characteristics. Sixth, even though our findings indicate an exciting scientific approach to further differentiate meniscus in health and disease, statistically significant differences between histological subgroups do not imply the ability to diagnose on qMRI parameters alone. With reproducibility of research being an increasingly important cornerstone of modern biomedical research, additional translational studies need to be conducted to confirm that the statistically significant differences observed in this study are of scientific and diagnostic relevance. In this regard, the apical meniscus zone should be primarily considered in efforts to differentiate the tissue’s functional status. Seventh, for biomechanical evaluation in terms of unconfined compression testing, an invasive preparation procedure was chosen that was characterized by resection of the top and bottom of the specimen cylinder. Even though biomechanical properties were certainly altered, the methodology is scientifically sound and biomechanically valid because the innermost third of the specimen, which largely determines compressive properties^53^, was entirely included in all specimens. Eighth, the use of a standard 3.0 T MRI device and the choice of sequence parameters was intended to facilitate eventual translation of our findings into clinical practice. While a scanner with higher field strength (e.g. 7.0 T or 9.4 T) would have provided better signal-to-noise ratio, such scanners have not (yet) seen widespread clinical application and relaxation times are different from those at 3.0 T^54^. We deliberately chose sequence parameters similar to those in earlier studies^55–59^ to render generalizability and transferability of the findings less related to actual sequence parameter settings.

In conclusion, this study is the first to systematically assess human meniscus functionality based on advanced quantitative MRI techniques and in relation to histological and biomechanical reference measures. Under standardized compressive loading, we determined different response-to-loading patterns across the meniscus zones that are related to histological degeneration. Changes in T1ρ relaxation and in the apical zone seem to indicate distinctly different loading-induced intra-tissue adaptations and may help to identify potential load transmission failure in degenerative joint disease. However, tissue hydration likely overpowered the contribution of biochemical alterations in this *in-vitro* study, which warrants further *in-vivo* studies.

## Methods

### Industry support

Philips Healthcare (Hamburg, Germany) supported this study by providing the T1ρ sequence. The authors had and have full control over the data and information submitted for publication.

### Study design

This study was designed as a prospective, *ex-vivo* observational imaging study on human lateral meniscus body samples obtained from patients undergoing total knee arthroplasty (TKA) at our institution. Approval from the relevant local Institutional Review Board (Ethical Committee, RWTH Aachen University, Germany [AZ-EK 157/13]) and individual written informed consent were obtained from all patients beforehand. All experiments were performed in accordance with relevant local guidelines and regulations.

### Preparation of meniscus samples

We harvested lateral meniscus body samples (n = 45) from patients undergoing elective total knee arthroplasty at our institution (n = 45, University Hospital Aachen, Germany). To avoid sample pooling, we only included one sample from each patient. Primary OA as determined radiographically (defined as Kellgren-Lawrence grades ≥ 2)^60^ was the inclusion criterion, while all forms of secondary OA, previous trauma to and/or surgery of the index joint and other bone and joint pathologies were the exclusion criteria. Overall, we included 23 left and 22 right knees of 24 male and 21 female patients with a mean age of 67.4 ± 10.9 years [range: 40–94 years].

We used dedicated software for statistical power analyses (G*Power freeware from Heinrich-Heine-University Düsseldorf, v3.1.9.4, URL: http://www.psychologie.hhu.de/arbeitsgruppen/allgemeine-psychologie-und-arbeitspsychologie/gpower.html)^61^ to determine minimum sample sizes. Based on earlier studies^22,23^, we estimated a minimum sample size of 39 for a statistical power of 0.9, a type-I error of 0.05, an effect size of 0.5, and three groups undergoing three measurements.

**Figure 5:**
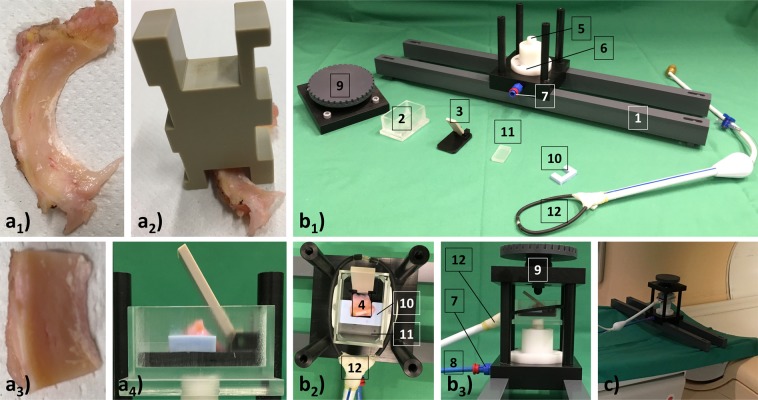
Preparation of meniscus samples and details of the MRI-compatible loading device. (**a**_**1**_) The entire lateral meniscus specimen (as obtained during surgery) was cleared of all adjacent capsular tissue. (**a**_**2**_) After identification of the meniscus body region, a dedicated cutting device was used to cut the specimen to standard length (i.e. anteroposterior diameter) of 15 mm. (**a**_**3**_) Eventually, meniscus samples of standard length and unaltered width (i.e. mediolateral diameter) were obtained. (**a**_**4**_) Prepared samples were placed into the lever device within the sample box for consecutive MRI measurements. (**b**) Photographs of the MRI-compatible loading device (**b**_**1**_: disassembled; **b**_**2**_: partially assembled; **b**_**3**_, (**c**): assembled) for pressure-controlled, quasi-static and torque-induced compressive loading of human meniscus samples under simultaneous MR imaging. For standardized positioning within the bore of the MRI scanner, the device was mounted on two parallel support beams (1) that were attached to the guiding rails of the exam table. Loading was brought about by pneumatically controlled upward displacement of the sample box (2) containing the lever device (3) and meniscus sample (4). The sample box was screwed onto the pneumatic piston (5) that had been laid out to generate (measured) forces in the range of 0–76N^62^ once the pressure chamber (6) was filled with air via the pressure connection port (7) and pressure lines (8). A half-sphere attached to the vertically adjustable cover screw contained within the device’s upper frame (9) then induced defined torque loading on the samples. Samples were confined by appropriately sized polyvinylsiloxane inlays (10) and PMMA plates (11) to prevent their lateral displacement upon loading. A dedicated modified prostate single-channel coil (12) that circumferentially comprised the sample box was used for imaging. c) Loading device in operation in the clinical 3.0-T MRI scanner.

After surgery, we prepared the specimens according to standard. First, we identified the meniscus body region of each surgical specimen and removed any adherent capsular soft tissues (Fig. 5a_1_). Second, we cut the specimen to standard anteroposterior diameter (i.e. length) of 15 mm using a cutting device that contained notches of variable depth (Fig. 5a_2_). Specimen width was not altered (Fig. 5a_3_). Tissue-marking dye (Polysciences, Warrington, US) was used to define the mediolateral imaging plane (from meniscus apex to base) for future reference. Samples were stored in sterile DMEM medium (Gibco-BRL, Gaithersburg, US) containing 100 U/ml penicillin, 100 μg/ml gentamycin and 1.25 U/ml amphotericin B (all from Gibco-BRL).

### Force-controlled compressive loading device

We applied standardized compressive loading on the meniscus samples using an MRI-compatible compressive loading device, which loads meniscus samples by means of torque^62^. In practical terms, we placed individual meniscus samples into the dedicated lever device within the sample box attached to the pneumatically driven sample tray (Fig. 5a_4_). By control of set pressure levels, the sample tray was displaced upwards towards a half-sphere attached to the vertically adjustable cover screw within the device’s upper frame (Fig. 5b). The upward movement was actuated by a pneumatic mechanism and resulted in a defined torque moment on the compression lever that conformed well to the sample’s wedge shape. Confining inlays (polyvinylsiloxane, Wirosil, Bego, Germany) circumferentially contained and supported the samples to prevent loading-induced sample displacement. We selected appropriately sized confining inlays to contain half of the meniscus base height. Additionally, polymethylmethacrylate (PMMA) plates of corresponding width were placed between inlay and sample box outlines. We loaded samples by control of pressure via an electronically actuated valve with high precision (±0.01 bar, Type: VPPM-6L-L-1-G18–0L6H-V1P-S1C1, Festo, Esslingen, Germany), a digital-to-analogue converter (Multifunction I/O USB-6001, National Instruments Corporation, Austin, USA) and customized software routines implemented in LabVIEW software (v2017, National Instruments Corporation, URL: https://www.ni.com/de-de/shop/labview.html). To this end, we connected the pneumatic mechanism via pressure lines (Festo) to the standard hospital pressure outlet providing pressure levels of up to 4.69 bar. The control components were located outside the MRI scanning room. During MRI measurements we set target pressures to three different levels, i.e. p_0_ = 0 bar (δ_0_, i.e. unloaded), p_1_ = 2 bar (δ_1_), and p_2_ = 4 bar (δ_2_). Earlier pressure-force calibration studies based on digital sensors (K-Scan 4000, Tekscan, Boston, USA) indicated that set pressure levels resulted in highly reproducible compressive forces of f_1_ = 37.1 ± 0.8 N (δ_1_) and f_2_ = 69.1 ± 1.1 N (δ_2_) on the half-spherical piston and resultant torques of t_1_ = 0.67 Nm (δ_1_) and t_2_ = 1.24 Nm (δ_2_) on the compression lever^62^.

### MRI measurements

Within 24 h after preparation, the meniscus samples underwent standardized MRI measurements in standard saline solution using a clinical 3.0 T MRI system (Achieva, Philips, The Netherlands) and the MRI-compatible compressive loading device. We selected appropriately sized polyvinylsiloxane inlays and PMMA plates to confine the sample circumferentially (Fig. 5). Next, we adjusted the height of the half-sphere (using the cover screw) to be in loose contact with the lever and the femoral meniscus surface. Imaging was performed using a modified single-channel (receive-only) prostate coil (BPX-30 endorectal coil, Medrad/Bayer, Germany) stripped off its inflatable balloon that circumferentially comprised the sample box. Radiofrequency pulses were applied via the scanner’s body coil and scout views were acquired to guide the imaging sections along the mediolateral plane for each sample and loading position individually. The imaging protocol consisted of Proton Density-weighted (PDw) sequences in the axial, coronal and sagittal orientation and T2, T1ρ, and T1 maps in the coronal orientation (Table 3). We imaged the samples at the reference configuration, i.e. δ_0_, to be followed by two consecutive pressure levels, i.e. δ_1_ and δ_2_. After each change in pressure level and before initiating the MRI measurements, we observed an equilibration period of 5 min. This time period was based on an earlier study that investigated stress relaxation as a function of physiological strains. Chia and Hull found equilibration in human meniscus to be complete after three to four minutes^39^. In other studies, considerably longer equilibration periods of up to 20 min were observed^35^.

**Table 3:** Acquisition Parameters of MR sequences.

	PDW	T1	T1ρ	T2
Sequence Type	Turbo-spin echo (2D)	Inversion-recovery (2D)	Spin-lock multi-gradient echo (3D)	Multi-spin echo (2D)
Orientation	ax, sag, cor	cor	cor	cor
Repetition Time [ms]	1500	3000	30	1500
Echo time [ms]	11.2	10.1	3.8	n × 8.4 (n = 1–6)
Turbo spin-echo factor	6	5	44	12
Field of view [mm]	62 × 62	62 × 62	52 × 52	52 × 52
Acquisition matrix	144 × 142	224 × 220	176 × 176	176 × 176
Reconstruction matrix	256 × 256	224 × 224	224 × 224	224 × 224
Flip angle [°]	90	90	11	90
Number of signal averages	2	1	4	2
Slices	10	1	7	1
Slice Thickness/Gap [mm]	1.0/1.5	2.0/n/a	3.2/3.2	2.0/n/a
Inversion times [ms]	n/a	150, 300, 500, 800, 1000, 1500	n/a	n/a
Spin-lock durations [ms]	n/a	n/a	0, 10, 20, 30, 40	n/a
Duration [min]	2.48	9.42	15.98	5.08

n/a - not applicable.

### Image processing

DT (a board-certified clinical MSK radiologist with 6 years of experience) carried out the manual segmentations of the meniscus samples. Using the morphological PDw images obtained along the mediolateral plane as references, segmentation masks of sample outlines were generated while excluding boundary pixels to eliminate partial volume effects. SN (clinical MSK radiologist, 6 years of experience) validated these masks against the processed T1, T1ρ, and T2 images. In addition to the entire sample outlines, further zonal ROIs were defined by automatic subdivision of segmented outlines using a custom-made routine implemented in MATLAB (MatlabR2018b, Natick, USA). First, this routine determined the sample’s maximum mediolateral diameter by projecting the segmented outline’s most peripheral pixels onto a horizontal line parallel to the lever device’s bottom. Second, after dividing this line into thirds, the division was back-projected onto the segmented outline to obtain the apical zone (I), intermediate zone (II), and outer zone (III) (consistent with the white-white, red-white, and white-white zones as defined histologically)^2^. Zonal ROIs were thus defined by these vertical thirds (as inner borders) and the segmented sample outline (as outer borders) (Fig. 1b).

### Biomechanical and histological reference evaluation

For biomechanical assessment, we harvested two cylindrical samples of 4 mm diameter from the red-red to the red-white transition, bilaterally adjacent to the mediolateral plane, using a 4mm-diameter biopsy punch (pfm-medical, Cologne, Germany) that was oriented perpendicular to the tibial surface. After resection of the femoral and tibial meniscus surfaces, we cut the punched-out core cylinders to standard height of 3 mm and carried out unconfined compression testing on a materials testing machine (Z2.5, Zwick/Roell, Ulm, Germany) equipped with a 20 mm wide rigid and impermeable piston. A tare load of 0.2 N was applied to maintain standardized interaction of the interfaces and mechanical equilibrium throughout the measurements. No additional preconditioning loads were applied. As in previous studies^63,64^, we applied a displacement rate of 0.0083 mm/sec and a strain of 100%.

The resultant stress-strain data obtained were fitted to a two-parameter exponential model^36,37^. Here, the strain energy Ψ is given by

1 $$\Psi =\frac{{\rm{c}}}{2{\rm{b}}}{[\exp ({\rm{b}}{\rm{\varepsilon }})-1]}^{2}$$

where b scales the stress response and c gives the degree of nonlinearity, while ε is strain. The resulting expression for the Elastic Modulus EM is given by the second derivative with respect to ε, hence

2 $$EM=c\,b\,\exp (b\varepsilon )$$

For each sample, non-linear optimizations were run in MATLAB to specifically fit the material parameters. Eventually, we determined the Elastic Modulus EM for the strains of 20% and 80% to obtain representative data from high and low strains and to limit the amount of data for subsequent multiple comparisons. Representative samples’ measured stress-strain curves and modelling predictions are included as supplementary material (Supplementary Figure 1).

For histological assessment, samples were fixed in paraformaldehyde (4%) for 7 days, embedded in paraffin, sectioned along the mediolateral plane, cut to 5-µm thick slices and stained with hematoxylin-eosin and Safranin O^13^. We used a digital light microscope (BZ-9000, Keyence, Osaka, Japan) to visualize the samples and merged six individual micrographs into one image per sample. Two investigators (SN [fellowship trained, 10 years of experience in histopathology]; LD [2 years of experience in histopathology]) graded the tissue status semi-quantitatively according to the Pauli classification^30^ in consensus. On a per-sample basis, final scores were discussed until consensus was reached. No measures of inter- or intra-rater variability were calculated. The Pauli classification assesses surface integrity for the femoral, tibial and inner surfaces (score 0–3 each, i.e. 0–9), cellularity (score 0–3), collagen organization and alignment (score 0–3), and matrix staining intensity (score 0–3). Accordingly, the Pauli sum score (range, 0–18) indicates either the absence of any signs of degeneration (score 0) or most severe degeneration (score 18). Based on Pauli sums scores, samples were grouped into Pauli grades, i.e. grossly intact (Pauli sum scores 0–4, Pauli Grade-I), early degenerative (5–9, Pauli Grade-II), moderately degenerative (10–14, Pauli Grade-III), and severely degenerative (15–18, Pauli Grade-IV). Due to limited sample sizes, Pauli Grade-III and -IV were collapsed as the Pauli Grades ≥ III subgroup.

### Statistical analysis

We performed the statistical analyses using GraphpadPrism (v6.0, San Diego, CA, USA). δ_0_ refers to the unloaded absolute qMRI parameter values, while δ_1_ and δ_2_ refer to the values under loading of 2 bar (δ_1_) and 4 bar (δ_2_). Consequently, relative changes in absolute qMRI parameter values at δ_1_ or δ_2_ versus δ_0_ are referred to as Δ_1_ or Δ_2_. For example, the relative change in T1ρ at δ_2_ vs. δ_0_ (connoted as T1ρΔ_2_) was calculated as T1ρΔ_2_ = (((T1ρ(δ_2_)/T1ρ(δ_0_)) − 1) * 100) [%]. After sample allocation, we performed group-wise comparisons using one-way ANOVA (for normally distributed data, e.g. Elastic Modulus) or the Kruskal-Wallis tests (for non-normally distributed data, e.g. absolute qMRI parameter values). Normality was tested using the D’Agostino-Pearson test. Longitudinal differences were assessed using the Friedman test followed by Dunn’s post-hoc tests. We evaluated differences in pixel numbers using repeated measures ANOVA, while we used unpaired t-tests for the ROI-specific relative changes. Correlations were quantified using Spearman’s correlation coefficient ρ. Normally distributed data are given as mean ± standard deviation and non-normally distributed data as median (interquartile range). Level of significance was set to p ≤ 0.005 to contain the number of statistically significant, yet scientifically (most likely) irrelevant findings.

## Supplementary information

Supplementary Information.

## Data Availability

The datasets generated and analyzed in this study are available from the corresponding author on reasonable request.
