# Supplementary material for: Functional MRI Mapping of Human Meniscus Functionality and its Relation to Degeneration
Source: Sci Rep. 2020 Feb 12;10:2499. doi: 10.1038/s41598-020-59573-4 (PMC7016001; doi:10.1038/s41598-020-59573-4)

Title: Functional MRI Mapping of Human Meniscus Functionality and Its Relation to Degeneration

Authors: Sven Nebelung, Lisa Dötsch, Dhaval Shah, Daniel Benjamin Abrar, Kevin Linka, Matthias Knobe, Philipp Sewerin, Johannes Thüring, Christiane Kuhl, Daniel Truhn

Journal: Scientific Reports

Supplementary Table 1 (online only)

	**Pauli Sum Score**	**EM (strain 0.2)**	**EM (strain 0.8)**	**T1 (δ_0)**	**T1ρ (δ_0)**	**T2 (δ_0)**	**T1 (δ_1)**	**T1ρ (δ_1)**	**T2 (δ_1)**	**T1 (δ_2)**	**T1ρ (δ_2)**	**T2 (δ_2)**
**Pauli Sum Score**		0.02 (0.89)	0.04 (0.82)	0.12 (0.43)	0.19 (0.21)	0.18 (0.25)	0.19 (0.21)	-0.09 (0.57)	0.14 (0.34)	0.00 (1.00)	-0.10 (0.52)	-0.01 (0.94)
**EM (strain 0.2)**	0.02 (0.89)		**0.77 (< 0.001)**	**-0.59 (< 0.001)**	**-0.43 (0.003)**	**-0.55 (< 0.001)**	**-0.54 (< 0.001)**	-0.38 (0.01)	**-0.50 (< 0.001)**	-0.35 (0.02)	-0.24 (0.12)	-0.12 (0.43)
**EM (strain 0.8)**	0.04 (0.82)	**0.77 (< 0.001)**		**-0.67 (< 0.001)**	**-0.57 (< 0.001)**	**-0.66 (< 0.001)**	**-0.60 (< 0.001)**	**-0.54 (< 0.001)**	**-0.57 (< 0.001)**	**-0.47 (0.001)**	-0.40 (0.01)	-0.17 (0.28)

Supplementary Figure 1 (online only)

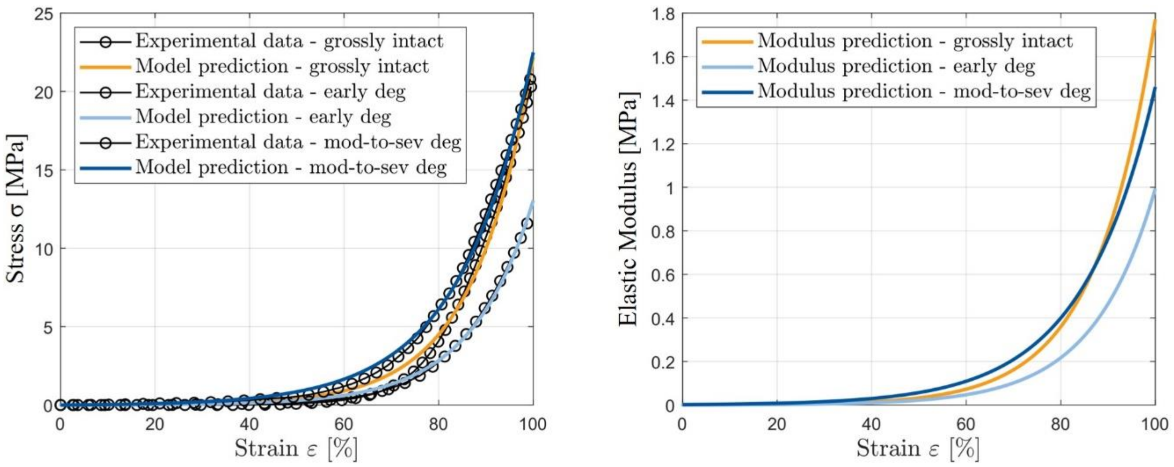

Supplement: Supplementary file 1 — Supplementary Information. [file 41598_2020_59573_MOESM1_ESM.pdf]
